# Retraction Note: CYPA promotes the progression and metastasis of serous ovarian cancer (SOC) in vitro and in vivo

**DOI:** 10.1186/s13048-023-01267-2

**Published:** 2023-08-25

**Authors:** Zhi-Ying Qi, Fang Wang, Ying-Ying Yue, Xue-Wang Guo, Rui-Meng Guo, Hong-Lin Li, Yan-Ying Xu

**Affiliations:** Department of Gynecolog, The Second Hospital of Tianjin Medical University, No.23 Pingjiang Road, Hexi District, Tianjin, 300211 China

**Retraction Note: J Ovarian Res 12, 118 (2019)**

https://doi.org/10.1186/s13048-019-0593-2

The Editors-in-Chief have retracted this article. After publication, concerns were raised regarding highly similar images between this article and a number of other articles that were submitted and published within a close time frame [1–5].

The authors have stated that the image duplication occurred due to the use of a shared platform, and that the original data could not be retrieved. Due to the number and severity of the duplications, the Editors-in-Chief no longer have confidence in the presented data.

All authors agree to this retraction.
